# Psychological, social, and motivational factors in persons who use drugs

**DOI:** 10.1186/s13011-020-00273-7

**Published:** 2020-04-29

**Authors:** Sana Shahrabadi, Amir Jalali, Rostam Jalali, Ali Gholami

**Affiliations:** 1 Department of Nursing, School of Nursing, Iran Young Researchers and Elite Club, Gorgan Branch, Islamic Azad University, Gorgan, Iran; 2 Substance Abuse Prevention Research Center, Research Institute for Health, Kermanshah University of Medical Sciences, Kermanshah, Iran; 3 Department of Nursing, School of Nursing and Midwifery, Kermanshah University of Medical Sciences, Kermanshah, Iran; 4 Department Anesthesiology, Clinical Development Research Center, Kermanshah University of Medical Sciences, Kermanshah, Iran

**Keywords:** Psychosocial functioning, Motivation for treatment, Persons who use drugs, Demographical variables

## Abstract

**Background:**

Persons who use drug need family and society’s support in the process of treatment and rehabilitation. Therefore, it is imperative to determine the psychological, social, and motivational factors that can help them in the treatment process. The present study was an attempt to determine the relationship between psychological, social, and motivational factors and the demographics of persons who use drugs (PWUD).

**Methods:**

An analytical cross-sectional study was carried out. TCU psychological functioning and motivation scales for the PWUD was first translated into Farsi and validated after securing permission from the copyright holder of the tool. Participants were 250 PWUDs under methadone therapy who were selected through convenient sampling. Before analyzing the collected data, validity and reliability of the tool were confirmed using confirmatory and exploratory factor analyses. Given the scale of demographical data, descriptive and analytic statistics were used to analyze the relationship between demographical variables and psychological, social, and motivational factors.

**Results:**

The results of exploratory and confirmatory factor analyses showed that out of 83 statements in the original questionnaire, 55 statements categorized into 11 aspects were usable for Iranian population. The results showed that gender, income, and marital status affect psychological functioning of the PWUD (*P* < 0.05). However, education level, place of residence, and type of drug and consumption did not have a significant relationship with social functioning of the participants (*p* > 0.05). There was a significant relationship between age, number of children, and history of using drug and psychological functioning of the participants (*P* < 0.01). The results showed that the demographics did not have a notable effect on the participants’ motivation for treatment; only marital status had a significant relationship with the participants’ treatment readiness (*P* < 0.05).

**Conclusion:**

As the results showed, the demographical variables could affect physical, psychological, and social functioning in the participants.

## Background

Drug dependence is one of the main psychosocial challenges in today society all around the world [1]. Every day, a large number of individuals are lured into drugs [2]. According to the World Health Organization, about 5.6% of 15–64 years old population in the world have used drugs at least once in their lives. About 31 million in the world suffer the disorders caused by using drugs [3]. In the case of Iran, the youth are at a high risk of developing drugs dependence due to cultural reasons, wrong beliefs, and neighboring one of the main producing countries of narcotic substances [4]. The immense losses in the form of lost lives and financial resources and the social outcomes (heavy costs, death, suicide, crime, divorce, sexually transmitted diseases like HIV and hepatitis) caused by using drugs are not negligible [5]. Shahbazi et al. (2017) reported that mortality rate of PWUDs in Iran was 38.4 individuals per one million, which is higher than the world average rate [6].

Abusive use of drugs in psychological patients is a prevalent issue that affects social and occupational performance of the individual [7]. Studies on abusive use of drug have shown that there is a direct relationship between abusive use of drugs and mental health [7, 8]. Developing drug dependence hinders fulfillment of social, spiritual, and emotional roles of the user at social and family levels; which causes problems for the society and family [9]. Psychosocial functioning is a key factor in the treatment and rehabilitation of PWUDs [10–12]. Personality factors and psychological ones in particular like happiness and self-esteem are of the main factors in the decision to quit [13, 14].

Social, psychological, and motivational factors can help the PWUD in making decision to quit drugs [12]. Physical, psychological, and motivational factors can help the PWUD in the treatment process and afterwards. In addition, demographical factors can be also useful [15]. As suggested by studies, the variables age, gender, marital status, and education level affect psychological functioning of the PWUD [16]. In addition, more than one half of the abusive users of drugs suffer psychological disorders [17]. On the other hand, perceiving the motivations of PWUD is very important for the treatment as it is knowing about the motivational factors when they want to quit [18]. Motivation has long been considered as a key factor in the treatment of risky behaviors like abusive use of alcoholic drinks and drugs. It is also highly important in the successful treatment of drugs abusive use [18, 19]. As showed by studies, there are very important motivational factors in the treatment and rehabilitation of PWUD [15, 20].

Given this introduction, it is essential to comprehend the relationship of demographical variables in the PWUD and their psychological, social, and motivational performance. Having a deep insight into the decisive factors, we can take more effective steps to alleviate the damages caused by abusive use of drugs through making more effective decisions to treat the patients. The demographic variables are related to psychosocial aspects, social functions, and tendency to treatment in PWUDs. Therefore, the present study was an attempt to survey the relationship between psychological, social, and motivational factors and some of the demographical factors.

## Methods

### Study design

A cross-sectional and descriptive-analytical study was carried out from Sep 2018 to June 2019.

### Participants

The study population consisted of all the PWUD visiting drug abuse clinics (22 clinics) located in Kermanshah City -Iran. Approximately, 100 clients had a file as outpatients in almost every clinic. Following [11, 21], 300 participants were selected through convenient sampling based on a set of inclusion criteria (only 250 questionnaire were fully completed and used in the study). For this purpose, the researcher would visit the clinic during business hours. All PWUD treated in the clinics with the inclusion criteria and willing to participate in the study were selected. Inclusion criteria were vising drug dependent treatment centers, both male and female; and willingness to participate in the study. The exclusion criterion was incomplete questionnaires.

### Tool translation

At first, modified Texas Christian University (TCU) psychological, social, and motivational performance questionnaire for drug users [22] was translated into Farsi through forward-backward method (Wild et al. [23]). Two independent translators translated the tool into Farsi and a translation team checked the translations to spot inconsistencies between the two translations. Two translators translated the draft translation back into English and inconsistencies between the original and translated versions were examined. The draft was designed and arranged as a standard questionnaire and provided to the PWUDs to comment on its understandability and clarity. The patients’ feedbacks were implemented on the questionnaire and vague and unfamiliar terms were corrected. Afterwards, content validity index (CVI), content validity ratio (CVR), and Kappa coefficient were obtained for the questionnaire. Then, data gathering process was started.

## Method

At first, the participants were briefed about the questionnaire and how to fill it and they signed a written letter of consent. Inclusion criteria were desire to participate, using herbal narcotic drugs, and at least 2 weeks under methadone therapy. In the tool validation stage, 10 experts gave their opinions about content validity of the tool, and to examine construct validity, the tool was provided to 250 PWUD. To examine content validity, Waltz and Bausell’s content validity index (CVI) was used and to examine correlation between the scores of tests and tools (test-retest reliability) Pearson’s correlation coefficient was used. In addition, Cronbach’s alpha was employed to check internal consistency of the tool. Exploratory and confirmatory factor analyses were used to check the construct validity. The relationship between demographical variables and psychological, social, and motivational performance was examined using independent t test, Pearson Correlation, and one-way ANOVA statistics.

### Instrument

In addition to a demographics form, the modified TCU psychological, social, and motivational functioning form was used. The latter is a self-rating form with 11 sub-scales and 88item that includes four psychological functioning scales, four social functioning scales, and three motivation scales [22]. The four psychological functioning scales (29 items) include composite measures of self-esteem (SE) - six items, depression (DP) - six items, anxiety (AX) – eight items, and decision-making confidence (DM) - nine items. The four social functioning scales (31 items) includes measures of childhood problems (CP) – eight items, hostility (HS) – eight items, risk-taking behavior (RT) – seven items, and social conformity (SC)- eight items. The three motivational scales (24 items) includes measures of problem recognition (PR)- nine items, desire for help (DH) – seven items, and treatment readiness (TR)- eight items. The scales each consist of 7 to 10 items, with items rated on a 5-point Likert scale (0 = strongly disagree, 1 = disagree, 2 = undecided, 3 = agree, 4 = strongly agree).

## Results

Mean age of the participants was 39.24 ± 11.73 and mean history of using drugs was 13.8 ± 11.04. Totally, 86.6% were men, 54.8% were married, 43.6% had an elementary level education, 40.4% had a high school diploma. Moreover, 29.2% had used opiate, 22.4% used heroin and crack heroin, and 48.4% used a combination of natural and industrial opiate. Additionally, 91.6% lived in urban area.

### Validation of the tool

The first step to check validity of the tool is content validity check. Waltz and Bausell’s CVI was employed to this end. As the results showed, the CVI and CVR were acceptable for all the statements and no statement was omitted at this stage. To examine reliability of the tool, test-retest technique was used through Pearson’s correlation coefficient, which yielded 0.875.

**Fig. 1:**
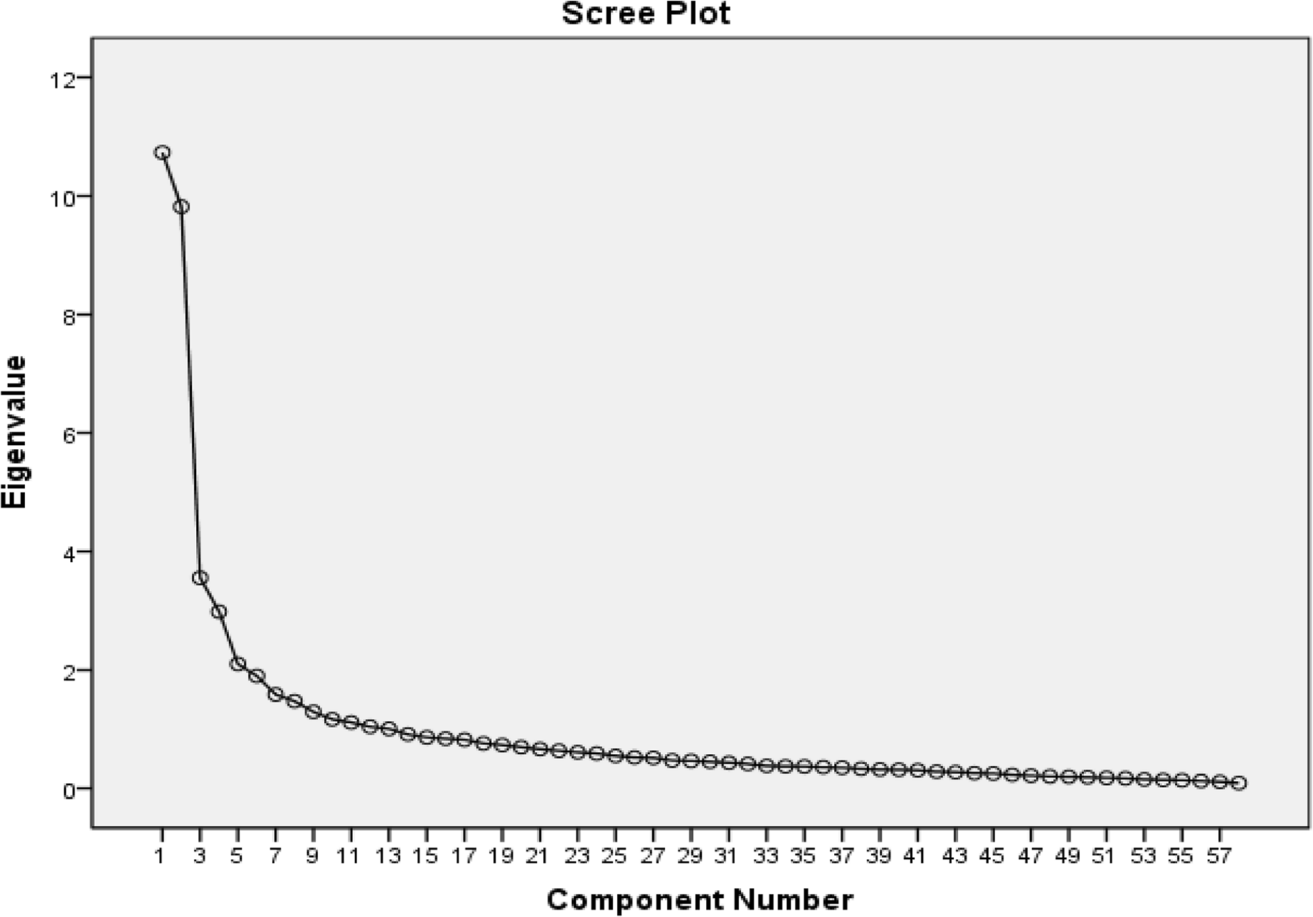
Scree Cattel plot of the extracted elements of the questionnaire

To examine construct validity, exploratory factor analysis was used followed by confirmatory factor analysis. In the former, correlation coefficients were examined for the statements to make sure that they are in an acceptable range. Kaiser Meyer Olkin (KMO) test and Bartlett’s test of sphericity were used to this end. Given that KMO = 0.858 > 0.7 and that Bartlett’s test was significant (Chi Square = 13,500/19, *P*-value < 0.01), the presumptions for using exploratory factor analysis on TCU questionnaire with 83 statements were met. Varimax vertical rotation was employed and the factors of which the specific value was above one were selected for exploratory factor analysis through principle components (PC) analysis. In this study, factors with eigenvalues greater than one were selected (Fig. 1).

In addition, commonality value of each statement was high (> 0.5) so that none of the questions were omitted in this stage. Still, factor load of the rotated variables showed that some of the variables had factor loading (> 0.3) on two factors at the same time and therefore, they were omitted. In this way, 24 statements (1, 2, 10, 12, 14, 15, 19, 23, 27, 28, 32, 35, 37, 47, 48, 51, 54, 56, 59, 68, 70, 74, 75, and 81) were omitted. In addition, statement No.26 was omitted because of low factor loading (< 0.3) on different factors. Thus, 57 statements remained in the study. Exploratory factor analysis was repeated using the main elements of the analysis and varimax rotation. Scree plot demonstrates factor analysis in SPSS so that 13 factors or elements are fitted for the final analysis (Table 1). The questions about each factor, name of each factor, and Cronbach’s alpha coefficients are listed in Table 2 to determine reliability of the elements. Exploratory factor analysis was completed with 11 factors and 56 statements.

**Table 1:** Factors extracted after exploratory analysis

Component	Initial Eigenvalues			Extraction Sums of Squared Loadings		
	Total	% of Variance	Cumulative %	Total	% of Variance	Cumulative %
1	10.735	18.509	18.509	6.882	11.866	11.866
2	9.818	16.928	35.437	6.312	10.882	22.748
3	3.554	6.128	41.565	5.403	9.316	32.064
4	2.981	5.140	46.704	3.918	6.755	38.819
5	2.101	3.623	50.328	3.615	6.233	45.052
6	1.900	3.276	53.604	3.417	5.892	50.944
7	1.586	2.735	56.339	1.870	3.225	54.169
8	1.473	2.540	58.879	1.672	2.884	57.052
9	1.293	2.229	61.107	1.441	2.484	59.536
10	1.166	2.011	63.118	1.409	2.429	61.965
11	1.113	1.919	65.038	1.349	2.325	64.290
12	1.041	1.795	66.833	1.255	2.164	66.454
13	1.005	1.733	68.566	1.225	2.111	68.566

**Table 2:** Exploratory factor analysis result

Variable	Element	Number of statements	Statements	T-value	λ	Cronbach’s alpha
Psychological functioning	Self-esteem (factor No.3)	5	3–4–5-6-7	14.5	0.83^***^	0.867
	Depression (factor No. 10)	3	8–9-11	16.32	0.91^***^	0.830
	Anxiety (factor No.7)	4	13–16–17-18	11.21	0.67^***^	0.749
	Decision-making confidence (factor No. 6)	5	20–21–22-24-25	2.8	−0.19^***^	0.908
Social functioning	Childhood problems (factor no. 10)	5	30–31–33-34-36	9.8	0.69^***^	0.739
	Hostility (factor No 11)	7	38–39–40-41-42-43-44	12.48	0.94^***^	0.941
	Risk-taking behavior (factor No. 8)	4	45–46–49-50	4.43	0.3^***^	0.773
	Social conformity (factor No. 5)	5	52–53–55-57-58	−6.4	−0.43^***^	0.754
Treatment motivation	Measures of problem recognition (factor no.2)	8	60–61–62-63-64-65-66-67	11.06	0.92^***^	0.927
	Desire for help (factor No.9)	4	69–71–72-73	5.86	0.63^***^	0.710
	Treatment readiness (factor No.4)	6	77–78–79-80-82-83	7.16	0.5^***^	0.887

***P < 0/001; **P < 0/01; *P < 0/05

First-order confirmatory factor analysis was used in this study in two steps. In the first step, factor loadings of the questionnaire questions were analyzed. Secondly, factor loadings of factors were analyzed (Table 3). Only the statement No. 46 had a low factor load (t = 0.26) and eliminated.

**Table 3:** Index of confirmatory factor analysis

Area	AGFI	CFI	NNFI	RMSEA	χ2/df
Psychological functioning	0.84	0.95	0.95	0.059	1.83
Social functioning	0.81	0.91	0.91	0.075	2.68
Treatment motivation	0.82	0.92	0.92	0.072	2.34

### Analysis of the relationships

As listed in Table 4, the variables gender, job, income, and marital status had a relationship with the psychological functioning of the PWUDs (*P* < 0.05). However, education level, place of residence, the way of using drugs, and the type of drugs did not have a significant relationship with one’s social functioning (*P* > 0.05).

**Table 4:** Comparison of the mean score and SD of the aspects of Psychological functioning in terms of the demographical variables

Variable		Self-esteem	Depression	Anxiety	Decision-making confidence	Psychological functioning
**Gender**^a^	**M**	**2.0 ± 25.95**	**2.1 ± 01.09**	**1.0 ± 78.88**	**1.0 ± 94.85**	**2.0 ± 0.64**
	**F**	**2.1 ± 54.02**	**2.0 ± 44.90**	**2.0 ± 7.91**	**1.0 ± 99.97**	**2.0 ± 26.63**
	**Sig.**	**0.110**	**0.015**	**0.081**	**0.758**	**0.030**
**Education**^b^	**Elementary level**	**2.1 ± 30.02**	**2.1 ± 1.02**	**1.0 ± 80.88**	**1.0 ± 80.85**	**1.0 ± 98.67**
	**High school**	**2.0 ± 25.95**	**2.1 ± 17.18**	**1.0 ± 88.95**	**1.0 ± 96.90**	**2.0 ± 7.68**
	**Higher education**	**2.0 ± 33.91**	**2.1 ± 1.00**	**1 ± 76.79**	**2.0 ± 22.80**	**2.0 ± 8.53**
	**Sig.**	**0.887**	**0.537**	**0.717**	**0.015**	**0.520**
**Place of residence**^a^	**Urban**	**2.0 ± 28.98**	**2.1 ± 16.07**	**2.0 ± 5.88**	**1.0 ± 94.88**	**2.0 ± 2.65**
	**Rural area**	**2.0 ± 33.78**	**2.1 ± 6.10**	**1.0 ± 80.92**	**2.0 ± 0.75**	**2.0 ± 13.54**
	**Sig.**	**0.791**	**0.700**	**0.245**	**0.736**	**0.375**
**Job**^b^	**Office employee**	**2.0 ± 37.95**	**1.1 ± 99.12**	**1.0 ± 79.89**	**2.0 ± 7.94**	**2 ± 5.66**
	**Housewife**	**2 ± 92.92**	**2 ± 71.82**	**2.0 ± 31.92**	**1.0 ± 77.96**	**2.0 ± 43.65**
	**Worker**	**2.0 ± 60.82**	**2.0 ± 31.98**	**1.0 ± 93.80**	**1.0 ± 77.77**	**2.0 ± 15.53**
	**Freelancer**	**2.0 ± 7.91**	**1.1 ± 88.07**	**1.0 ± 77.91**	**2.0 ± 5.82**	**1.0 ± 94.63**
	**Unemployed**	**1.1 ± 88.31**	**2.1 ± 16.29**	**1.0 ± 41.75**	**1.1 ± 66.07**	**1.0 ± 78.76**
	**Retired**	**1.0 ± 48.36**	**1.0 ± 28.89**	**1.0 ± 20.60**	**2.0 ± 8.76**	**1.0 ± 51.56**
	**University student**	**2.0 ± 90.14**	**2.0 ± 17.24**	**2.0 ± 38.18**	**2.0 ± 0.05**	**2.0 ± 36.1**
	**Sig.**	**0.000**	**0.009**	**0.023**	**0.264**	**0.004**
**Marital status**^b^	**Unmarried**	**2.0 ± 44.93**	**2.1 ± 29.12**	**1.0 ± 98.84**	**1.0 ± 92.84**	**2.0 ± 16.62**
	**Married**	**2.0 ± 22.91**	**1.1 ± 87.03**	**1.0 ± 73.88**	**2.0 ± 2.90**	**1.0 ± 96.61**
	**Divorced**	**2.1 ± 61.13**	**2.1 ± 41.14**	**2.1 ± 0.01**	**1.0 ± 80.71**	**2.0 ± 20.76**
	**Widow**	**1.0 ± 31.78**	**2.0 ± 12.70**	**1.0 ± 36.55**	**1.0 ± 58.95**	**1.0 ± 59.59**
	**Sig.**	**0.001**	**0.013**	**0.046**	**0.276**	**0.008**
**Income**^b^	**Low**	**1.0 ± 48.89**	**2.1 ± 28.02**	**1.0 ± 95.85**	**1.0 ± 96.82**	**1.0 ± 91.59**
	**Moderate**	**1.1 ± 99.04**	**1.1 ± 74.10**	**1.0 ± 61.91**	**1.0 ± 83.94**	**1.0 ± 79.70**
	**High**	**1.0 ± 82.96**	**1.0 ± 52.98**	**1.0 ± 51.92**	**2.0 ± 36.84**	**1.0 ± 80.61**
	**Sig.**	**0.000**	**0.000**	**0.007**	**0.053**	**0.000**
**Way of using**^b^	**Smoking**	**2.1 ± 14.10**	**1.1 ± 92.07**	**1.0 ± 83.95**	**1.0 ± 94.89**	**1.0 ± 96.75**
	**Injection**	**2.0 ± 70.93**	**2.0 ± 58.94**	**2.0 ± 9.57**	**1.0 ± 67.38**	**2.0 ± 26.54**
	**Oral**	**2.0 ± 29.97**	**1.1 ± 98.16**	**1.0 ± 69.75**	**2.0 ± 23.92**	**2.0 ± 5.58**
	**Inhaling**	**2.0 ± 56.88**	**2.0 ± 45.93**	**2.0 ± 2.84**	**1.0 ± 62.72**	**2.0 ± 16.53**
	**Mixed**	**2.0 ± 33.95**	**2.1 ± 29.11**	**1.0 ± 92.88**	**1.0 ± 79.82**	**2.0 ± 8.79**
	**Sig.**	**0.759**	**0.365**	**0.691**	**0.063**	**0.821**
**Type of drug**^a^	**Only one type**	**2.0 ± 12.76**	**1.0 ± 98.75**	**1.1 ± 78.12**	**2.0 ± 30.91**	**2.0 ± 4.93**
	**Mixed**	**2.0 ± 3.93**	**2.1 ± 15.06**	**2.0 ± 3.88**	**2.0 ± 10.63**	**2.0 ± 7.93**
	Sig.	**0.367**	**0.158**	**0.143**	**0.287**	**0.542**

^a^independent sample test

^b^One-way ANOVA

Table 5 lists the Pearson correlation coefficients to examine the relationship between demographical variables, psychological functioning, and its aspects.

**Table 5:** The relationship (Pearson correlation coefficients) between demographical variables and Psychological functionin

Variable	Self-esteem	Depression	Anxiety	Decision-making confidence	Psychological functioning
Age	−0.277**	0.226**	0.162*	−0.016	−0.249**
Number of children	−0.196**	0.198**	0.250**	−0.076	−.0268**
The experience of drug use	−.0.201**	0.166**	0.208**	−.007	−0.219**
Age of first experience of drugs	−0.027	−0.081	−0.021	−0.02	−0.042

*P < 0.05; ** *P* < 0.01

As listed in Table 5, there is a significant relationship between age, number children, and the history of using drugs and psychological functioning (*P* < 0.01). In other words, with an increase in the demographical variable, a decrease in psychological functioning takes place. However, there was no relationship between the age of first experience of drugs and psychological functioning (*p* = 0.513). Table 6 compares mean score and SD of the aspects of social functioning in terms of demographical variables.

**Table 6:** Comparison of the mean score and SD of the aspects of social functioning in terms of the demographical variables

Variable		Childhood problems	Violence	Risk-taking behavior	Social conformity	Social functioning
Gender^a^	M	1.0 ± 58.71	1.1 ± 60.10	2.0 ± 51.84	2.0 ± 13.79	1.0 ± 95.45
	F	1.0 ± 46.054	1.1 ± 58.09	2.1 ± 47.02	2.0 ± 00.82	1.0 ± 92.41
	Sig.	**0.363**	**0.934**	**0.331**	**0.316**	**0.674**
Education^b^	Elementary level	1.0 ± 60.69	1.1 ± 63.10	2.0 ± 55.85	2.0 ± 17.77	1.0 ± 99.44
	High school	1.0 ± 55.74	1.1 ± 50.06	2.0 ± 52.90	2.0 ± 4.84	1.0 ± 90.48
	Higher education	1.0 ± 50.61	1.1 ± 68.17	2.0 ± 52.85	2.0 ± 10.76	1.0 ± 95.39
	Sig.	**0.700**	**0.611**	**0.963**	**0.526**	**0.432**
Place of residence^a^	Urban	1.0 ± 54.69	1.1 ± 54.11	2.0 ± 51.87	2.0 ± 11.80	1.0 ± 92.44
	Rural area	1.0 ± 79.61	2.0 ± 18.81	20 ± 78.76	2.0 ± 13.74	2.0 ± 22.40
	Sig.	**0.086**	**0.011**	**0.142**	**0.872**	**0.004**
Job^b^	Office employee	1.0 ± 47.64	1.0 ± 45.94	2.0 ± 72.77	2.0 ± 28.79	1.0 ± 98.38
	Housewife	1.0 ± 50.60	1.1 ± 65.12	2.0 ± 73.84	1.0 ± 85.72	1.0 ± 93.35
	Worker	1.0 ± 74.74	1.1 ± 86.18	2.0 ± 48.77	2.0 ± 07.73	2.0 ± 4.50
	Freelancer	1.0 ± 55.69	1.1 ± 56.09	2.0 ± 54.87	2.0 ± 16.83	1.0 ± 95.44
	Unemployed	1.0 ± 40.77	1.1 ± 56.34	2.1 ± 4.07	1.0 ± 82.84	1.0 ± 70.48
	Retired	0.0 ± 94.43	0.0 ± 60.60	1.1 ± 93.14	1.0 ± 92.67	1.0 ± 35.29
	University student	2.0 ± 10.14	2.0 ± 14.20	2.0 ± 67.94	1.0 ± 80.29	2.0 ± 18.15
	Sig.	**0.131**	**0.191**	**0.072**	**0.260**	**0.012**
Marital status^b^	Unmarried	1.0 ± 65.71	1.1 ± 74.10	2.0 ± 43.91	2.0 ± 00.75	1.0 ± 96.42
	Married	1.0 ± 54.67	1.1 ± 47.06	2.0 ± 70.79	2.0 ± 28.77	2.0 ± 00.43
	Divorced	1.0 ± 57.77	1.1 ± 82.28	2.0 ± 30.90	1.0 ± 81.82	1.0 ± 88.47
	Widow	1.0 ± 20.55	1.0 ± 30.93	1.0 ± 78.89	1.0 ± 47.73	1.0 ± 44.44
	Sig.	**0.224**	**0.121**	**0.001**	**0.000**	**0.001**
Income^b^	Low	1.0 ± 64.73	1.1 ± 76.10	2.0 ± 50.86	2.0 ± 5.74	1.0 ± 98.45
	Moderate	1.0 ± 49.62	1.1 ± 46.04	2.0 ± 56.88	2.0 ± 11.87	1.0 ± 91.44
	High	1.0 ± 17.44	0.0 ± 76.92	2.0 ± 73.80	2.0 ± 60.79	1.0 ± 82.38
	Sig.	**0.010**	**0.000**	**0.484**	**0.013**	**0.0478**
Way of using^b^	Smoking	1.0 ± 53.70	1.1 ± 59.07	2.0 ± 54.90	2.0 ± 00.80	1.0 ± 91.49
	Injection	2.0 ± 40.73	2.1 ± 45.00	1.0 ± 78.87	1.0 ± 70.76	2.0 ± 0.8.37
	Oral	1.0 ± 36.58	1.1 ± 22.12	2.0 ± 90.79	2.0 ± 49.83	1.0 ± 99.37
	Inhaling	1.0 ± 91.73	2.1 ± 38.00	2.0 ± 09.92	1.0 ± 98.58	2.0 ± 08.62
	Mixed	1.0 ± 70.71	1.1 ± 95.02	2.0 ± 33.66	2.0 ± 07.94	2.0 ± 01.45
	Sig.	**0.074**	**0.084**	**0.012**	**0.121**	**0.431**
Type of drug^a^	Only one type	1.0 ± 68.75	1.1 ± 86.11	2.0 ± 63.89	1.0 ± 70.78	1.0 ± 96.49
	Mixed	1.0 ± 75.59	1.0 ± 80.87	2.1 ± 66.02	1.0 ± 49.69	1.0 ± 92.41
	Sig.	**0.42**	**0.538**	**0.654**	**0.041**	**0.739**

^a^independent sample test

^b^One-way ANOVA

As the findings showed, place of residence, job, income, and marital status had a relationship with social functioning of the patients (*P* < 0.05). However, gender (*p* = 0.674), education level (*p* = 0.432), way of using drug (*p* = 0.431), and type of drug (*p* = 0.739) did not have a significant relationship with social functioning. Table 7 lists Pearson correlation coefficients for the relationship between demographical variables, psychological functioning, and its aspects.

**Table 7:** The relationship (Pearson correlation coefficients) between demographical variables and social functioning

Variable	Childhood problems	Violence	Risk-taking behavior	Social conformity	Social functioning
Age	−0.084	0.143*	0.060	0.089	−0.052
Number of children	−.098	−.097	0.002	0.067	−0.067
The experience of drug use	−0.064	−0.093	−.0.012	0.028	−0.075
Age of first experience of drugs	−0.040	0.036	0.074	0.071	0.030

*P < 0.05; ** *P* < 0.0

As listed in Table 7, there is a negative significant relationship between age and violence in the patients (*p* < 0.01). In other words, with an increase in age, violence declines in the patients. There was no significant relationship between other demographical variables and social functioning (*p* > 0.05). As listed in Table 8, none of the demographical variables are related to the motivation for treatment in the subjects (*p* > 0.5). Only marital status was significantly related to treatment readiness. So that, the divorces/widows had more motivation to quit. In addition, the type of drug has a significant relationship with treatment readiness (*P* < 0.05); so that patients who use only one type of drug have more desire for treatment.

**Table 8:** Comparing mean score and SD of the aspects of Treatment motivation in terms of demographical variables

Variable		Measures of problem recognition	Desire to seek help	Treatment readiness	Treatment motivation
Gender^a^	M	2.0 ± 96.87	2.0 ± 49.70	2.0 ± 37.95	2.0 ± 60.66
	F	2.0 ± 89.80	2.0 ± 40.62	2.0 ± 54.85	2.0 ± 61.65
	Sig.	**0.679**	**0.442**	**0.285**	**0.957**
Education^b^	Elementary level	2.0 ± 86.84	2.1 ± 39.02	2.0 ± 36.93	2.0 ± 54.66
	High school	3.0 ± 4.85	2.1 ± 62.18	2.0 ± 35.99	2.0 ± 67.64
	Higher education	2.0 ± 97.92	2.1 ± 40.00	2.0 ± 53.84	2.0 ± 63.70
	Sig.	**0.331**	**0.056**	**0.506**	**0.367**
Place of residence ^a^	Urban	2.0 ± 97.85	2.1 ± 46.07	2.0 ± 39.94	2.0 ± 60.67
	Rural area	2.0 ± 75.94	2.1 ± 62.10	2.0 ± 43.88	2.0 ± 60.57
	Sig.	**0.321**	**0.260**	**0.839**	**0.967**
Job^b^	Office employee	2.0 ± 98.97	2.0 ± 45.79	2.0 ± 43.96	2.0 ± 62.78
	Housewife	3.0 ± 6.64	2.0 ± 45.53	2.0 ± 63.85	2.0 ± 71.58
	Worker	2.0 ± 90.83	2.0 ± 50.76	2.0 ± 49.89	2.0 ± 63.69
	Freelancer	2.0 ± 91.91	2.0 ± 49.65	2.0 ± 34.94	2.0 ± 58.64
	Unemployed	3.0 ± 16.71	2.0 ± 51.65	2.1 ± 24.11	2.0 ± 64.23
	Retired	2.0 ± 50.40	2.0 ± 35.70	1.0 ± 77.98	2.0 ± 21.58
	University student	3.0 ± 50.35	2.1 ± 0.06	2.0 ± 33.71	2.0 ± 61.71
	Sig.	**0.682**	**0.967**	**0.548**	**0.847**
Marital status^b^	Unmarried	3.0 ± 4.84	2.0 ± 56.68	2.0 ± 42.95	2.0 ± 68.65
	Married	2.0 ± 90.86	2.0 ± 47.67	2.0 ± 41.90	2.0 ± 60.65
	Divorced	3.0 ± 6.96	2.0 ± 28.75	2.0 ± 56.88	2.0 ± 63.74
	Widow	2.0 ± 56.91	2.0 ± 50.77	1.1 ± 44.04	2.0 ± 17.60
	Sig.	**0.262**	**0.320**	**0.005**	**0.122**
Income^b^	Low	2.0 ± 98.83	2.0 ± 53.65	2.0 ± 48.89	2.0 ± 66.63
	Moderate	2.0 ± 96.89	2.0 ± 42.76	2.1 ± 19.02	2.0 ± 53.68
	High	2.0 ± 69.99	2.0 ± 23.70	2.0 ± 36.92	2.0 ± 42.78
	Sig.	**0.37**	**0.130**	**0.088**	**0.146**
Way of using^b^	Smoking	2.0 ± 99.90	2.0 ± 51.74	2.1 ± 37.03	2.0 ± 62.69
	Injection	3.0 ± 2.81	2.0 ± 56.46	2.0 ± 75.65	2.0 ± 78.49
	Oral	3.0 ± 3.89	2.0 ± 42.70	2.0 ± 54.91	2.0 ± 66.66
	Inhaling	3.0 ± 0.69	20 ± 59.74	2 ± 86.67	2.0 ± 82.59
	Mixed	2.0 ± 95.95	2.1 ± 50.11	2.0 ± 33.99	2.0 ± 59.92
	Sig.	**0.942**	**0.980**	**0.275**	**0.696**
Type of drug^a^	Only one type	20 ± 78.76	3.0 ± 1.70	2.0 ± 35.88	2.0 ± 71.74
	Mixed	3.0 ± 10.93	2.0 ± 89.98	2.0 ± 3.83	20 ± 67.69
	Sig.	**0.087**	**0.158**	**0.049**	**0.542**

^a^independent sample test

^b^One-way ANOVA

As listed in Table 9, there is a negative relationship between number of children and motivation for treatment (*p* < 0.05, *r* = − 0.139). That is, with an increase in the number of children, the motivation in patients declines.

**Table 9:** The relationship (Pearson correlation coefficients) between demographical variables and Treatment motivation

Variable	Measures of problem recognition	Desire to seek help	Treatment readiness	Treatment motivation
Age	−0.067	−0.080	−0.037	−0.019
Number of children	−0.130*	−0.075	−0.120	−0.139*
The experience of drug use	−0.110	−0.055	−0.085	−0.107
Age of first experience of drugs	−0.004	0.130*	0.042	0.63

* *P* < 0.05, ** *P* < 0.01

## Discussion

Perceiving the effects of demographical variables on psychological, social, and motivational performance of PWUDs may lead to better treatment protocols. The relationship between the demographical variables in PWUDs and their social, psychological, and motivational functioning was examined. Gender had an effect on depression score of the participants – i.e. an aspect of psychological functioning- so that it was higher in females than males. In general, gender affected the psychological functioning of PWUDs so that female drug addicts, being more sensitive than male, were more vulnerable to psychological damages. This finding was more consistent with other studies [9, 24]. However, the effect of gender on social functioning of the PWUDs was not significant.

Education level of the participants affected the confidence in decision-making -i.e. an aspect of psychological functioning. In general, however, the effect of education level on psychological and social functioning was not significant. Place of residence was another variable under study and it affected the level of violent behavior -i.e. an aspect of social functioning. That is, urban dwellers were less violent than those living in rural areas. One probable reason for this is that the latter group live in a smaller community and they feel more pressure by their society for being a drug addict. In general, and consistent with Poudel et al. (2016) [25], the small sample group of rural dwellers and the considerable level of interactions between rural and urban areas in Iran can explain this finding.

Job was another factor in psychological and social functioning. That is, those who had a job had a better social and psychological functioning than those without a job. The results showed that job affected the participants’ self-esteem, depression, and anxiety (psychological functioning) and risk-taking attitudes (social functioning). This is consistent with other studies [3, 9]. Many studies have shown that having a decent job is a factor in enabling the PWUD [24] and it can improve their physical and psychological functioning [3, 5, 24, 26].

Marital status affected self-esteem, depression, and anxiety (psychological functioning); so that the unmarried individuals had a better psychological functioning. This finding is consistent with other studies like [9, 16]. Risk-taking attitude and social conformity in the married individual were higher than the others; which is consistent with Gyawali and Sarkar (2016) [27].

Individuals with a higher income had higher self-esteem and confidence in decision-making. They also had lower anxiety and depression (psychological functioning). Moreover, the PWUDs with higher income had fewer childhood problems, were less violent, and were more socially adaptable. To explain this, a better economic condition attenuates social problems and improves the quality of life [17]. Socioeconomic condition of family and proper family support [16] can be effective in self-esteem, happiness [13], and even the quality of life [18] as they play a key role in treatment and prevention of relapse [9].

There was a negative relationship between age and psychological functioning of PWUD. That is, the older individuals had more psychological functioning problems compared to the younger clients. In addition, the level of violence was lower in the older PWUD. Number of children and the history of using drugs were of other variables effective in psychological functioning of the subjects. These two variables had a negative relationship with psychological functioning of the participants. Drug users with more children or a longer history of using drugs had a lower psychological functioning. These findings are consistent with Poudel et al. (2016) [25].

Demographical variables did not have a notable effect on motivation for treatment. Only marital status had a significant relationship with treatment readiness; that is, unmarried individuals had more motivation for treatment. One probable reason is that unmarried addicts might have higher hopes for starting a new life. This finding is consistent with German et al. (2006) [19]. Another explanation for this might be the fact that married PWUD have to spend more time and money on the welfare of their children as the first priority of the family. In addition, desire for treatment was higher in the subjects who only used one type of drug; which is consistent with Targowski et al. (2004) [18]. Another reason for this finding is that PWUD who only use one type drug have a higher hope for rehabilitation. It appears, however, that the demographical variables are not very effective in the motivation for treatment. Probably, other factors including inner, personal, and family factors are more effective in the motivation for treatment.

Sampling and selection of clients was faced with difficulties. Stratified random sampling was the best method for the study, however, due to inclusion criteria and the unwillingness of many clients to participate in the study, this sampling method was not possible. Also a few of female clients participated in the study after extensive explanations and assurance of confidentiality. At the analysis stage, 50 questionnaires that were not completely filled in were omitted.

## Conclusion

Some demographical variables like gender, education level, job, marital state, age, education level, income, number of children, and the experience of drug use were related to psychological functioning. In addition, place of residence, job, marital state, age, income and type of drugs use were related to social functioning. Marital status, number of children, age of the first experience of drugs and type of drugs use were related to the motivation for treatment. Thus, the demographic variables have an effect on the process of treatment and rehabilitation in PWUDs.

## Data Availability

The datasets used and analyzed during the current study are available from the corresponding author on reasonable request.

## Abbreviation

CVI: Content Validity Index
CVR: Content Validity Ratio
PWUD: Person(s) Who Use(s) Drugs
KMO: Kaiser Meyer Olkin
TCU: Texas Cristian University
PC: Principle Components
KUMS: Kermanshah University of Medical Sciences
